# Serum proteomics analysis and comparisons using iTRAQ in the progression of hepatitis B

**DOI:** 10.3892/etm.2013.1310

**Published:** 2013-09-18

**Authors:** LIANG PENG, JING LIU, YANG-MEI LI, ZHAN-LIAN HUANG, PEI-PEI WANG, YU-RONG GU, YU-BAO ZHENG, ZHI-LIANG GAO

**Affiliations:** 1 Departments of Infectious Diseases, Third Affiliated Hospital of Sun Yat-sen University, Guangzhou, Guangdong 510630, P.R. China; 2 Traditional Chinese Medicine, Third Affiliated Hospital of Sun Yat-sen University, Guangzhou, Guangdong 510630, P.R. China

**Keywords:** serum, proteomics, isobaric tags for relative and absolute quantitation, hepatitis B

## Abstract

The aim of this study was to analyze the changes in serum protein levels in the progression of hepatitis B using isobaric tags for relative and absolute quantitation (iTRAQ) analysis, in addition to comparing the serum protein levels of patients with chronic hepatitis B (CHB), patients with hepatitis B virus-induced acute-on-chronic liver failure (HBV-induced ACLF) and normal individuals. Protein analysis was performed on 15 serum samples using iTRAQ. The study population included healthy controls (n=5), patients with CHB (n=5) and patients with HBV-induced ACLF (n=5). Western blotting was used to verify the results in an additional nine serum samples from healthy controls, patients with CHB and patients with HBV-induced ACLF (n=3, respectively). Using iTRAQ analysis, 16 different serum proteins with ≥1.5-fold differences in expression levels were identified in the patients with CHB and ACLF compared with the healthy controls. Five of those proteins, C-reactive protein precursor, hemoglobin β chain variant Hb S-Wake, apolipoprotein J precursor, platelet factor 4 precursor and vitronectin, which demonstrated the greatest differences in their expression levels and the most significant correlation with liver diseases, were subsequently verified using western blotting. The western blotting results were consistent with the results from the iTRAQ. Two of the five proteins are not classified by biological process, and the biological functions of all the proteins in HBV-induced ACLF remain unclear. This preliminary study demonstrated that a correlation between the expression of various serum proteins and the different pathogenetic conditions induced by HBV may exist. The analysis of a larger number of samples is required to identify potential protein biomarkers that may be involved in the pathogenesis and progression of hepatitis B.

## Introduction

In China, the hepatitis B surface antigen (HBsAg) seropositive rate for the general population (between 1 and 59 years of age) is 7.18% (1). Globally, there are ~93 million individuals with hepatitis B virus (HBV) infections, 20 million of which are chronic (2). Hepatitis B is the most common risk factor for liver cirrhosis and hepatocellular carcinoma (HCC), and the mortality of HBV-induced acute-on-chronic liver failure (ACLF) may exceed 60%. HBV infections are not easy to cure, and the mechanisms of the virus are unclear, particularly with regard to protein expression and regulation function in the pathogenic process.

Proteomics analysis is a powerful technology used in a myriad of studies, including those focused on liver diseases (3–7). Isobaric tags for relative and absolute quantitation (iTRAQ), as a quantitative method, is a common tool in proteomics and has been suggested to be as sensitive as (or more sensitive than) the differential in gel electrophoresis (DIGE) technique (8). Specifically, the iTRAQ method has been used to study a variety of diseases and has been shown to be effective and accurate (4,5,9).

The purpose of this study was to analyze serum protein levels using iTRAQ in normal controls, as well as patients with chronic hepatitis B (CHB) and HBV-induced ACLF, and to verify those results using western blotting. The ultimate aim was to identify the differences in serum protein levels that were closely associated with the progression of hepatitis B and HBV-induced ACLF. The results of this study may provide crucial information regarding viral mechanisms and the pathogenic process.

## Materials and methods

### Patients and specimens

Serum samples from healthy individuals and patients with CHB and HBV-induced ACLF were obtained from the Department of Infectious Diseases and the Department of Traditional Chinese Medicine of the Third Affiliated Hospital of Sun Yat-sen University (Guangzhou, China) (Tables I and II). HBV-induced ACLF was diagnosed using the previously described criteria (10,11). The exclusion criteria included pregnant and lactating females, patients that had been treated with antivirals or immunomodulatory therapy within the previous six months, the presence of other factors causing active liver diseases (e.g. autoimmune, drug-induced liver, alcoholic liver and inherited metabolic liver diseases), concomitant human immunodeficiency virus (HIV) infection or congenital immune deficiency diseases, liver cancer or other malignancies, severe diabetes, autoimmune diseases, other important organ dysfunctions (e.g. kidney dysfunction), concomitant infection (e.g. fever, leukocytosis or neutrophilia; manifestations of abdominal, biliary tract or lung infection) or other serious comorbidities (e.g. hepatic encephalopathy and gastrointestinal bleeding).

The study protocol conformed to the Ethical Guidelines of the 1975 Declaration of Helsinki and was approved by the appropriate Institutional Review Committee of Third Affiliated Hospital of Sun Yat-sen University (Guangzhou, China) and the Bureau of Health (Guangdong, China). Informed patient consent was obtained prior to participation in this study.

### Sample preparation and protein extraction

Plasma was fractionated with ProteinMiner Protein Enrichment Small-Capacity kit, Cat# 163-3006, (ProteinMiner ; Bio-Rad Laboratories, Hercules, CA, USA) according to the manufacturer’s instructions. Protein solutions were reduced for 1 h at 56°C with 10 mM dithiothreitol (DTT) and were cysteine-blocked with 55 mM iodoacetamide (IAA) at room temperature for 10 min. Each sample was precipitated with four-times the volume of cold acetone. The total protein concentration was determined using the Bradford assay.

### Protein digestion and peptide tagging

Protein solutions [100 *μ*g; 5 mg/ml in 0.5 M triethyl ammonium bicarbonate (TEAB) containing 0.1% sodium dodecyl sulfate, pH 8.5]were digested for 24 h with 10 *μ*g L-1-(4-tosylamido)-2-phenylethyl tosylphenylalanyl chloromethyl ketone (TPCK)-treated trypsin. Each peptide solution was labeled for 3 h at room temperature using an iTRAQ reagent that had been reconstituted in 70 liters of ethanol, in accordance with the iTRAQ Reagents Multiplex kit instructions (Applied Biosystems, Foster City, CA, USA). The reaction was terminated by adding MilliQ water, and the samples were labeled with 114, 115, 116 and 117 mass-tagged iTRAQ reagents.

### Strong cation exchange chromatography (SCX)

SCX was performed to remove excess iTRAQ reagents and interfering substances for the mass analysis. The labeled peptides were then dried in a vacuum centrifuge and resuspended in 200 ml Buffer A, prior to being loaded on a Phenomenex Luna 55 *μ*m SCX 100A column [250×4.60 mm (length × internal diameter), 5 *μ*m; Phenomenex, Inc., Torrance, CA, USA] on an Agilent 1100 HPLC unit (Agilent Technologies, Santa Clara, CA, USA). Buffer A consisted of 10 mM KH_2_PO_4_ and 25% acetonitrile at pH 3.0, while Buffer B consisted of 10 mM KH_2_PO_4_, 25% acetonitrile and 2 M KCl at pH 3.0. The 60 min gradient comprised 100% Buffer A for 30 min, 0–5% Buffer B for 1 min, 5–30% Buffer B for 15 min, 30–50% Buffer B for 5 min, 50% Buffer B for 4 min and 50–100% Buffer A for 5 min. Thirteen fractions were collected using a Foxy JR Fraction Collector (Dionex Corp., Sunnyvale, CA, USA). Fractions 2 and 3 were pooled according to the chromatogram profile, based on the peak intensity. All fractions were then dried in a vacuum concentrator and stored at −20°C, prior to further analysis using mass spectrometry.

### Nano liquid chromatography (LC) coupled to quadrupole time-of-flight (Q-TOF) with tandem mass spectrometry (MS/MS)

Peptides from the SCX fractions were dissolved in 0.3 *μ*l 100% formic acid and diluted to 5 *μ*l in 0.05% trifluoroacetic acid (TFA). The peptides were loaded onto a micrOTOF-Q II-nano LC system (Bruker Daltonics, Inc., Billerica, MA, USA) and mass spectra were acquired in the 250–1,600 m/z range every second for 60 min.

### Database screening

Data were processed using BioTools software (Bruker Daltonics, Inc.). The files were subsequently submitted to an in-house Mascot server (Matrix Science Ltd., London, UK) for database screening. The data were screened against the human sequence database [National Center for Biotechnology Information non-redundant (NCBInr)]. The search was performed using trypsin as a specific enzyme. A maximum of one missed cleavage was permitted and oxidation (M), iTRAQ 4 plex (K) and iTRAQ 4 plex (N-term) were selected as variable modifications. The data obtained on the micrOTOF-Q were searched with a peptide mass tolerance of 10 ppm and a fragment mass tolerance of 0.8 Da. Protein ratios were normalized using the overall median ratio for all the peptides in the sample for each separate ratio in every individual experiment. The ratio for a given protein was calculated by taking the average of all the peptide ratios that identified the protein. The final list of protein ratios was an average of the protein ratios of the three experiments and consisted only of proteins discovered in ≥2 of the three experiments. Function definitions of the variable protein contents were searched for using the following two websites: http://www.uniprot.org/ and http://www.ncbi.nlm.nih.gov/.

### Western blotting

Five proteins [C-reactive protein (CRP) precursor, hemoglobin β chain variant Hb S-Wake, apolipo-protein J precursor, platelet factor 4 precursor and vitronectin (VN)], which demonstrated the greatest differences in their expression levels and the most significant correlation with liver diseases, were chosen and verified by western blotting using western blotting kits (Forevergen, Guangzhou, China). Briefly, the protein lysates were separated by polyacrylamide gel electrophoresis, transferred to a polyvinylidene difluoride (PVDF) membrane and subjected to immunoblotting with the following antibodies: Anti-apolipoprotein J precursor antibody (ab16077; dilution, 1:2,000; Abcam, Cambridge, UK), anti-hemoglobin β (sc-21757; dilution, 1:500; Santa Cruz Biotechnology, Inc., Santa Cruz, CA, USA), anti-CRP precursor (ab32412; dilution, 1:1,000; Abcam), anti-platelet factor 4 precursor (AB1488P; dilution, 1:5,000; Millipore, Billerica, MA, USA) and anti-VN (ab11591; dilution, 1:500; Abcam) at 4°C overnight. After washing, the membranes were incubated with horseradish peroxidase-conjugated secondary antibodies and visualized using an enhanced chemiluminescence system (ECL; Forevergen, Guangzhou, China).

## Results

Tables I and II summarize the clinical characteristics of the patients from whom the samples were collected. iTRAQ identified 16 different proteins that had ≥1.5-fold differences in expression level between the patients with HBV-induced ACLF and CHB, respectively, and the healthy controls (Table III). Protein quantification software, based on the relative content of the isotopic reporter group and using m/z 114 as a reference, showed significant results (P≤0.05). Some identified protein was lost in the corresponding reporter group, giving no quantification information. The specific information and re-identification will be published in a future study.

**Figure 1.**
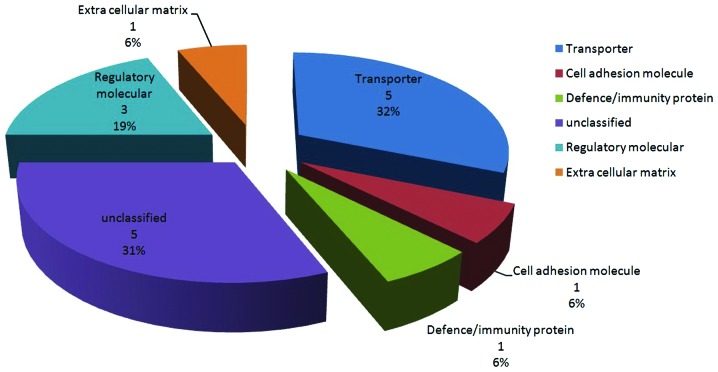
Classification of 16 serum proteins into different categories, based on protein function. The six categories were transporter, cell adhesion molecule, defence/immunity protein, regulatory molecular, extracellular matrix and unclassified.

**Figure 2.**
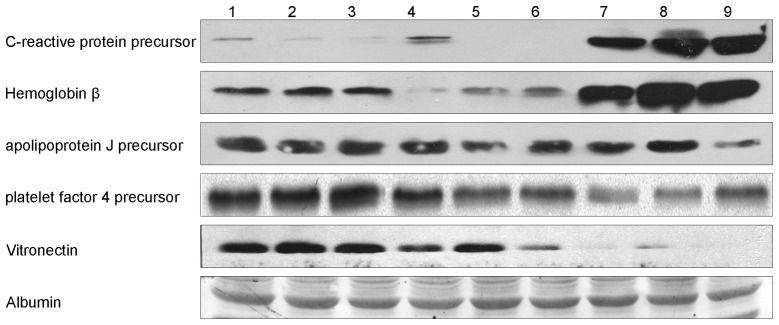
Results of verification using western blotting. Lanes 1–3, normal serum; 4–6, serum from patients with chronic hepatitis B (CHB); 7–9, serum from patients with hepatitis B virus (HBV)-induced acute-on-chronic liver failure (ACLF).

The 16 proteins that were identified using iTRAQ were classified into six categories, based on protein function (Fig. 1). The five proteins that demonstrated the greatest differences in their expression and the most significant correlation with liver diseases were verified using western blotting: CRP precursor, hemoglobin β chain variant Hb S-Wake, apolipoprotein J precursor, platelet factor 4 precursor and VN (Fig. 2 and Table IV). Two of the five proteins were not classified according to biological processes (apolipoprotein J precursor and platelet factor 4 precursor).

## Discussion

Proteomics research is a potentially useful and effective tool for studying pathogenesis, establishing prognosis and determining treatment outcomes in a variety of diseases. In the field of CHB, proteomics is not widely performed, presumably due to the mechanism of CHB being so complicated. The aim of this study was to describe the changes in serum protein levels in patients with CHB and HBV-induced ACLF, respectively, compared with healthy controls using iTRAQ and western blotting. The authors hypothesized that this approach had the potential to ultimately be beneficial for the identification of proteins that were important in the progression of HBV infections.

In the present study, 16 unique proteins were identified in patients with CHB and those with HBV-induced ACLF that had ≥1.5-fold differences in expression compared with those in the healthy controls. Those proteins belonged to six different categories based on protein function, while two out of the five proteins that were analyzed using western blotting were not classified according to biological processes (apolipoprotein J precursor and platelet factor 4 precursor). The biological functions of the remaining proteins in HBV infection and progression that were analyzed have not yet been elucidated; however, a number of theories are discussed in the following section.

CRP, synthesized in the liver, is an acute phase protein that is rapidly identified in the plasma in cases of infection or tissue damage. CRP is able to activate complement and enhance phagocytosis to facilitate the removal of pathogenic microorganisms and injured, necrotic and apoptotic cells. Therefore, CRP is important in natural immunity (12,13). Originally, CRP was considered to be a nonspecific biomarker of inflammation; however, following ~10 years of investigation, it has been demonstrated that CRP participates in inflammation and atherosclerosis directly and has been indicated to be a prognostic factor and a risk factor (14). In this study, it was revealed that levels of the precursor of CRP were decreased in the patients with CHB and markedly increased in the patients with ACLF. Nearly identical changes were observed for the hemoglobin β chain variant Hb S-Wake, which has been implicated in in orphan diseases (15), although not in liver diseases.

The apolipoprotein J precursor is important in cell aging and tumorigenesis with apolipoprotein J, lipid transfer inhibitor protein, complement C3d, corticosteroid-binding globulin and apolipoprotein L1 (16,17). These latter five markers of fibrosis are secreted in the blood and show consistent changes with the increasing stage of fibrosis when compared with the markers used in the FibroTest and enhanced liver fibrosis (ELF), Hepascore and FIBROSpect tests. In the present study, the levels of apolipoprotein J precursor decreased in the progression of HBV from CHB to ACLF. Therefore, further studies, with a larger number of samples, are required in order to better establish the role of apolipoprotein J precursor as a potential biomarker for HBV progression.

Platelet factor 4 is chemotactic for numerous cell types. It also functions as an inhibitor of hematopoiesis, angiogenesis and T-cell function, which may be used as a biomarker for establishing a prognosis in severe acute respiratory syndrome (18). Changes in platelet morphology, with accompanying increases in megathrombocyte fraction, may occur in chronic liver diseases, and thrombocytes are activated in chronic liver diseases and liver cirrhosis. Platelet sensitivity to stimuli in patients with liver cirrhosis has been demonstrated to be higher than in healthy controls (19) and it was indicated that platelet factor 4 was activated and upregulated in chronic hepatitis and liver cirrhosis. In the present study, levels of platelet factor 4 precursor decreased in the progression of hepatitis B to liver failure. This result suggests that further studies are required to investigate the potential of platelet factor 4 precursor as a biomarker in the progression of CHB.

VN is another protein that commonly features in studies on liver disease. It is a multifunctional plasma glycoprotein produced by hepatocytes that is detectable in plasma and the extracellular matrix. Glycosylation of VN modulates multi-merization and collagen binding in liver regeneration (20) and alterations in VN glycosylation modulate substrate adhesion to rat hepatic stellate cells (HSCs), which is responsible for matrix restructuring (21). Immunoblotting data identified increases in VN levels in cirrhotic livers, and VN immunoreactivity has been shown to be markedly increased in the cirrhotic liver matrix, irrespective of the apparent reduction in plasma VN. VN immunoreactivity in the liver may be considered to be a marker of fibrosis, particularly for chronic/mature fibrosis, paralleling previous observations concerning the enhanced orcein staining of cirrhotic septa (22). These results suggest that VN may be important in the progression of liver disease and/or in hepatic fibrosis through its collagen-binding domain I, and that VN levels are likely to be higher in patients with chronic hepatitis, liver cirrhosis and liver cancer than in controls (20–23). In the present study, VN levels decreased in the progression of hepatitis B (from CHB to HBV-induced ACLF). The observation of decreased VN levels was consistent with two other studies (24,25). In one of these studies, it was demonstrated that, in chronic liver disease, the concentration of plasma VN was significantly lower than in healthy controls and was associated with the severity of liver disease. Notably, levels of VN in the liver tissue were significantly increased in patients with chronic liver disease compared with those in normal controls. These results suggested that VN was deposited in injured tissue during the repair process and functioned as an adhesive protein (24). In the other study, it was demonstrated that there were lower levels of plasma VN in chronic liver disease, which was possibly due to a decreased synthesis, deposition in injured tissues or a combination of the two (25). The same group of authors also revealed that decreased levels of plasma VN and fibronectin (FN) and increased levels of serum laminin (LM) P1 in patients with chronic liver diseases were associated with hepatic dysfunction, and that changes in the levels of the glycoproteins involved in cell attachment were significant in the development of hepatic fibrosis in patients with chronic liver diseases (25). Based on the accumulating information on VN and liver fibrosis, it appears that VN requires further study.

In the present study, the authors aimed to describe changes in serum protein levels in patients with liver dysfunction induced by hepatitis B and patients with severe liver damage (ACLF). The purpose of this was to specifically identify one or more proteins that are able serve as biomarkers for predicting a pathogenic condition and for prognostic purposes, similar to α-fetoprotein (AFP) for hepatocellular carcinoma (HCC). It was not possible in this study to obtain a series of serum samples from individual patients as they progressed through the various stages of hepatitis B infection to severe liver damage (ACLF). However, using iTRAQ and western blotting, several candidate proteins were identified (namely apolipoprotein J precursor, platelet factor 4 precursor and VN) that warrant further study using a larger number of samples.

## Figures and Tables

**Table I.** Clinical characteristics of the 15 serum samples for iTRAQ testing.

No.	Source of serum	Gender	Age (years)	HBsAg	HBsAb	HBeAg	HBeAb	HBcAb	AST (14.5–40.0 U/l)	ALT (3–35 U/l)	TBIL (4.0–23.9 *μ*mol/l)	PT (11.0–14.5 sec)	HBV DNA (IU/ml)
1	Healthy control	Male	34	−	+	−	−	−	21.0	31	13.8	12.3	<100
2	Healthy control	Male	28	−	+	−	−	−	31.0	30	20.1	13.1	<100
3	Healthy control	Male	36	−	+	−	−	−	15.0	24	20.1	13.5	<100
4	Healthy control	Male	27	−	+	−	−	−	19.5	22	22.0	11.8	<100
5	Healthy control	Male	31	−	+	−	−	−	17.5	20	13.3	12.7	<100
6	CHB patient	Male	48	+	−	+	−	+	45.0	82	20.7	12.1	8.03×10^7^
7	CHB patient	Male	28	+	−	+	−	+	38.0	84	22.4	14.1	1.76×10^6^
8	CHB patient	Male	32	+	−	+	−	+	39.0	43	15.9	13.5	8.18×10^6^
9	CHB patient	Male	24	+	−	+	−	+	36.0	38	21.6	12.9	1.02×10^6^
10	CHB patient	Male	22	+	−	+	−	+	49.0	57	18.7	14.2	3.21×10^6^
11	HBV-induced ACLF patient	Male	39	+	−	−	+	+	26.0	35	463.5	25.5	6.52×10^4^
12	HBV-induced ACLF patient	Male	51	+	−	+	−	+	78.0	65	312.5	35.2	1.78×10^6^
13	HBV-induced ACLF patient	Male	46	+	−	+	−	+	101.0	46	556.7	29.1	3.55×10^6^
14	HBV-induced ACLF patient	Male	55	+	−	+	−	+	58.0	110	636.8	32.3	4.21×10^5^
15	HBV-induced ACLF patient	Male	32	+	−	−	+	+	115.0	78	482.5	28.8	6.17×10^5^

iTRAQ, isobaric tags for relative and absolute quantitation; HBsAg, hepatitis B surface antigen; HBsAb, hepatitis B surface antibody; HBeAg, hepatitis B e antigen; HBeAb, hepatitis B e antibody; HBcAg, hepatitis B core antigen; AST, aspartate aminotransferase; ALT, alanine aminotransferase; TBIL, total bilirubin; PT, prothrombin time; HBV, hepatitis B virus; CHB, chronic hepatitis B; ACLF, acute-on-chronic liver failure.

**Table II.** Clinical characteristics of the nine serum samples for western blotting verification.

No.	Source of serum	Gender	Age (years)	HBsAg	HBsAb	HBeAg	HBeAb	HBcAb	AST (14.5–40.0 U/l)	ALT (3–35 U/l)	TBIL (4.0–23.9 *μ*mol/l)	PT (11.0–14.5 sec)	HBV DNA (IU/ml)
1	Healthy control	Male	36	−	+	−	−	−	21.0	31.0	13.8	12.3	<100
2	Healthy control	Male	30	−	+	−	−	−	15.0	24.0	20.1	11.1	<100
3	Healthy control	Male	30	−	+	−	−	−	19.2	28.6	15.6	12.8	<100
4	CHB patient	Male	41	+	−	+	−	+	32.0	75.4	20.5	11.3	1.88×10^6^
5	CHB patient	Male	31	+	−	+	−	+	45.0	47.0	19.7	14.5	7.24×10^6^
6	CHB patient	Male	37	+	−	+	−	+	36.0	41.0	23.6	11.9	3.22×10^6^
7	HBV-induced ACLF patient	Male	21	+	−	+	−	+	104.5	68.0	278.9	27.3	5.66×10^6^
8	HBV-induced ACLF patient	Male	37	+	−	+	−	+	67.0	54.0	342.1	29.7	6.58×10^6^
9	HBV-induced ACLF patient	Male	43	+	−	−	+	+	36.0	78.0	521.4	31.5	7.33×10^5^

HBsAg, hepatitis B surface antigen; HBsAb, hepatitis B surface antibody; HBeAg, hepatitis B e antigen; HBeAb, hepatitis B e antibody; HBcAg, hepatitis B core antigen; AST, aspartate aminotransferase; ALT, alanine aminotransferase; TBIL, total bilirubin; PT, prothrombin time; HBV, hepatitis B virus; CHB, chronic hepatitis B; ACLF, acute-on-chronic liver failure.

**Table III.** The 16 proteins that were identified using iTRAQ as having ≥1.5-fold differences in their expression level between patients with HBV-induced ACLF and CHB, respectively, and healthy controls.

Accession no.	Protein name	Biological processes	Molecular function	Protein function	Levels in patients with CHB versus controls	Levels in HBV-induced ACLF patients versus controls
Q7TMA5	Apolipoprotein B-100 precursor	Cholesterol and lipid metabolism, lipid transport	Heparin binding	Transporter	Upregulated 1.62-fold	Upregulated 1.32-fold
P04004	Vitronectin	Cell adhesion	Heparin and integrin binding	Cell adhesion molecule	Downregulated 1.23-fold	Downregulated 2.14-fold
P01764	Monoclonal IgM antibody heavy chain	Immune response	Antigen binding	Defense/immunity protein	Upregulated 1.41-fold	Upregulated 1.87-fold
Unclassified	Unnamed protein product	Unclassified	Unclassified	Unclassified	Downregulated 1.23-fold	Downregulated 2.14-fold
P02768	Serum albumin preproprotein	Transport	Binding capacity	Transporter	Upregulated 1.86-fold	Upregulated 2.83-fold
Unclassified	Apolipoprotein J precursor	Unclassified	Unclassified	Unclassified	Downregulated 1.23-fold	Downregulated 2.00-fold
Unclassified	Unnamed protein product	Unclassified	Unclassified	Unclassified	Upregulated 1.07-fold	Upregulated 2.00-fold
D3DUX1	Serpin peptidase inhibitor, clade A	Regulatory	Serine-type endopeptidase inhibitor activity	Regulatory molecular	Normal	Upregulated 2.64-fold
P00450	Ceruloplasmin (ferroxidase), isoform CRA_b	Copper and iron transport	Chaperone binding, ferroxidase activity	Transporter	Upregulated 2.30-fold	Upregulated 2.64-fold
P07203	Glutathione peroxidase	UV protection, anti apoptosis, cell redox homeostasis and glutathione metabolic process	Oxidoreductase, peroxidase	Regulatory molecular	Downregulated 1.07-fold	Upregulated 2.46-fold
P68871	Hemoglobin β chain variant Hb S-Wake	Oxygen transport	Hypotensive agent, vasoactive	Transporter	Downregulated 1.07-fold	Upregulated 9.19-fold
B3KUE5	Phospholipid transfer protein, isoform CRA_c	Regulatory	Lipid binding	Regulatory molecular	Upregulated 1.32-fold	Upregulated 4.92-fold
Unclassified	Platelet factor 4 precursor	Unclassified	Unclassified	Unclassified	Downregulated 1.15-fold	Downregulated 1.87-fold
P19095	C reactive protein precursor	Acute phase response	Sugar binding	Unclassified	Downregulated 2.46-fold	Upregulated 4.59-fold
P18428	Lipopolysaccharide binding protein, isoform CRA_a	Lipid transport, transport	Antibiotic, antimicrobial	Transporter	Upregulated 1.07-fold	Upregulated 1.62-fold
P60709	Actin, β	Cellular component movement	ATP and kinesin binding	Extracellular matrix	Downregulated 1.23-fold	Upregulated 4.29-fold

iTRAQ, isobaric tags for relative and absolute quantitation; HBV, hepatitis B virus; ACLF, acute-on-chronic liver failure; CHB, chronic hepatitis B; IgM, immunoglobulin M.

**Table IV.** Five serum proteins with the greatest differences in their expression levels and the most significant correlation with liver diseases, as verified using western blotting.

Accession no.	Protein name	Biological processes	Molecular function	Protein function	Levels in CHB patients versus controls	Levels in HBV-induced ACLF patients versus controls
P19095	C reactive protein precursor	Acute phase response	Sugar binding	Unclassified	Downregulated 2.46 fold	Upregulated 4.59 fold
P68871	Hemoglobin β chain variant Hb S-Wake	Oxygen transport	Hypotensive agent, vasoactive	Transporter	Downregulated 1.07-fold	Upregulated 9.19-fold
Unclassified	Apolipoprotein J precursor	Unclassified	Unclassified	Unclassified	Downregulated 1.23-fold	Downregulated 2.00-fold
Unclassified	Platelet factor 4 precursor	Unclassified	Unclassified	Unclassified	Downregulated 1.15-fold	Downregulated 1.87-fold
P04004	Vitronectin	Cell adhesion	Heparin and integrin binding	Cell adhesion molecule	Downregulated 1.23-fold	Downregulated 2.14-fold

CHB, chronic hepatitis B; HBV, hepatitis B virus; ACLF, acute-on-chronic liver failure.
